# Paroxysmal nocturnal hemoglobinuria masquerading as hemolytic uremic syndrome: a Case Report

**DOI:** 10.3389/fmed.2025.1553168

**Published:** 2025-07-31

**Authors:** Menghan Gao, Bo Liu, Jianping Yao, Fuhan Huang

**Affiliations:** ^1^Department of Nephrology, Huzhou Central Hospital, Fifth School of Clinical Medicine of Zhejiang Chinese Medical University, Affiliated Central Hospital of Huzhou University, Huzhou, Zhejiang, China; ^2^Department of Endocrinology, Huzhou Central Hospital, Fifth School of Clinical Medicine of Zhejiang Chinese Medical University, Affiliated Central Hospital of Huzhou University, Huzhou, Zhejiang, China

**Keywords:** acute kidney injury, case report, hemolytic uremic syndrome, hemosiderin deposition, misdiagnosis, paroxysmal nocturnal hemoglobinuria

## Abstract

Paroxysmal nocturnal hemoglobinuria (PNH) is a rare acquired disorder of intravascular hemolysis caused by a somatic mutation in the gene responsible for glycosylphosphatidylinositol (GPI)-anchored complement regulatory proteins. This mutation leads to the production of abnormal blood cell clones lacking CD55 and CD59. PNH can result in renal damage. The challenge of early identification and diagnosis leads to misdiagnosis as other intravascular hemolytic conditions. This paper presents a case that began with fever, diarrhea, and acute renal failure, initially misdiagnosed as atypical hemolytic uremic syndrome (aHUS) but later confirmed as PNH through renal biopsy and related diagnostic tests. After treatment, the patient’s renal function recovered, and anemia improved. Intravascular hemolysis is a prominent feature common to both PNH and HUS. They exhibit similar clinical manifestations, which pose a challenge for differential diagnosis. Unlike previous reports, the patient in this case denied any history of hematologic disorders, which made the diagnosis more challenging.

## Introduction

Paroxysmal nocturnal hemoglobinuria (PNH) is a rare form of intravascular hemolysis resulting from an acquired deficiency of complement regulatory glycoproteins (1). Classic PNH is characterized by chronic intravascular hemolysis and hemoglobinuria, which can lead to renal damage. However, due to its low incidence and nonspecific clinical presentation, it is often misdiagnosed as other conditions. Hemolytic uremic syndrome (HUS) comprises a heterogeneous group of disorders that share a common pathological feature: thrombotic microangiopathy (TMA), which is classically characterized by a triad of non-immune microangiopathic hemolytic anemia, thrombocytopenia, and organ dysfunction, such as acute kidney injury (AKI) (2). We report a case of PNH that presented with the onset of fever and diarrhea, along with anemia, thrombocytopenia, and AKI, which was initially misdiagnosed as atypical hemolytic uremic syndrome (aHUS). The final diagnosis was confirmed through flow cytometry, fluorescence-labeled modified aerolysin (FLAER) testing, and kidney biopsy. PNH and HUS share certain clinical similarities, which pose a challenge for differential diagnosis, particularly in the absence of a prior history of hematologic disorders. When acute renal failure is accompanied by anemia, it is essential to consider hematologic disorders as a potential underlying cause.

## Case presentation

A 50-year-old male presented with a four-day history of fever, diarrhea, and low back pain. The patient reported that the fever occurred following physical exertion and was accompanied by chills, approximately 10 episodes of watery diarrhea per day, nausea, and a reduced appetite. The patient reported no significant past medical history and denied any personal or family history of renal or hematologic disorders, as well as any history of drug use or alcohol consumption. On admission, his temperature was 38°C, blood pressure was 132/92 mmHg, and pulse rate was 88 beats per minute. Physical examination revealed tenderness on bilateral renal percussion and mild edema of the lower extremities. The remainder of the physical and systemic examinations, including cardiorespiratory and dermatological assessments, were unremarkable. Laboratory investigations upon admission (Table 1) revealed anemia (hemoglobin, 77 g/L; schistocytes, 2%), thrombocytopenia (platelet count, 93 × 10^9^/L), inflammation (c-reactive protein 28.5 mg/L, procalcitonin 0.71 ng/mL), and renal impairment (urine protein 2+, urine occult blood 3+, serum creatinine 1,121 μmol/L). Ultrasound of the urinary system revealed diffuse bilateral renal enlargement, with no evidence of deep vein thrombosis. The normal ADAMTS13 activity excluded thrombotic thrombocytopenic purpura (TTP). The clinical diagnosis initially favored aHUS. The patient was treated with hemodialysis, cephalosporin-based antibiotic therapy, glucocorticoids (methylprednisolone 80 mg per day administered intravenously), and fresh frozen plasma transfusion. The therapeutic efficacy was reflected in the improvement of symptoms (reduction in low back pain, increased urine output, and resolution of fever and diarrhea), laboratory findings (hemoglobin stabilized at 65 g/L and progressive decline in serum creatinine), and imaging studies (computed tomography indicated a reduction in the size of the previously enlarged kidneys).

**Table 1:** Photomicrograph of kidney biopsy.

Urinalysis	Observed value	Reference range	Biochemistry	Observed value	Reference range
Occult blood	3+	-	Total protein	60.8	65.0–85.0 g/L
Glucose	±	-	Albumin	35.2	40.0–55.0 g/L
RBC	0–2	<2 /HP	AST	38	15.0–40.0 U/L
WBC	2.3	<13.2 /μL	ALT	20.2	9.0–50.0 U/L
24 h urinary protein	0.4	<0.15 g	LDH	2233.8	<248.2 U/L
			Total bilirubin	15	1.7–22 μmol/L
**Complete blood counts**			Uric acid	642.6	149–417 mmol/L
WBC	4.8	3.5–9.5 *10^9^/L	BUN	33.56	3.20–7.14 mmol/L
RBC	2.18	4.3–5.8 *10^12^/L	Creatinine	1,121	44–132.6 μmol/L
Hemoglobin	77	130–175 g/L	Sodium	138	137–147 mmol/L
Platelets	93	125–350 *10^9^/L	Potassium	4.2	3.5–5.3 mmol/L
Reticulocytes	1.5	0.5–1.5%	Chloride	108.4	99–110 mmol/L
Fragmented red cells	2	%	Calcium	1.88	2.17–2.75 mmol/L
**Coagulation**			Phosphorus	1.86	0.81–1.45 mmol/L
PT	12.9	9.7–14.1 s	Magnesium	1.04	0.70–1.10 mmol/L
APTT	27.6	25.1–36.5 s	**Immunology**		
D-dimer	0.86	<0.5 mg/L	CRP	28.5	<10 mg/L
**PNH cell types**			ANA	-	-
Red blood cells	7.8	%	Anti-dsDNA Ab	-	-
Granulocytes	66.9	%	ANCA	-	-
Monocyte	14.1	%	C3	1.02	0.79–1.52 g/L
**ADAMTS13 activity**	130.91	42.16–126.37%	C4	0.15	0.16–0.38 g/L
**Blood culture**	-	-	IgG	10.9	7.23–16.85 g/L
**Stool culture**	-	-	IgA	1.65	0.69–3.82 g/L
			IgM	0.54	0.63–2.77 g/L

**Figure 1:**
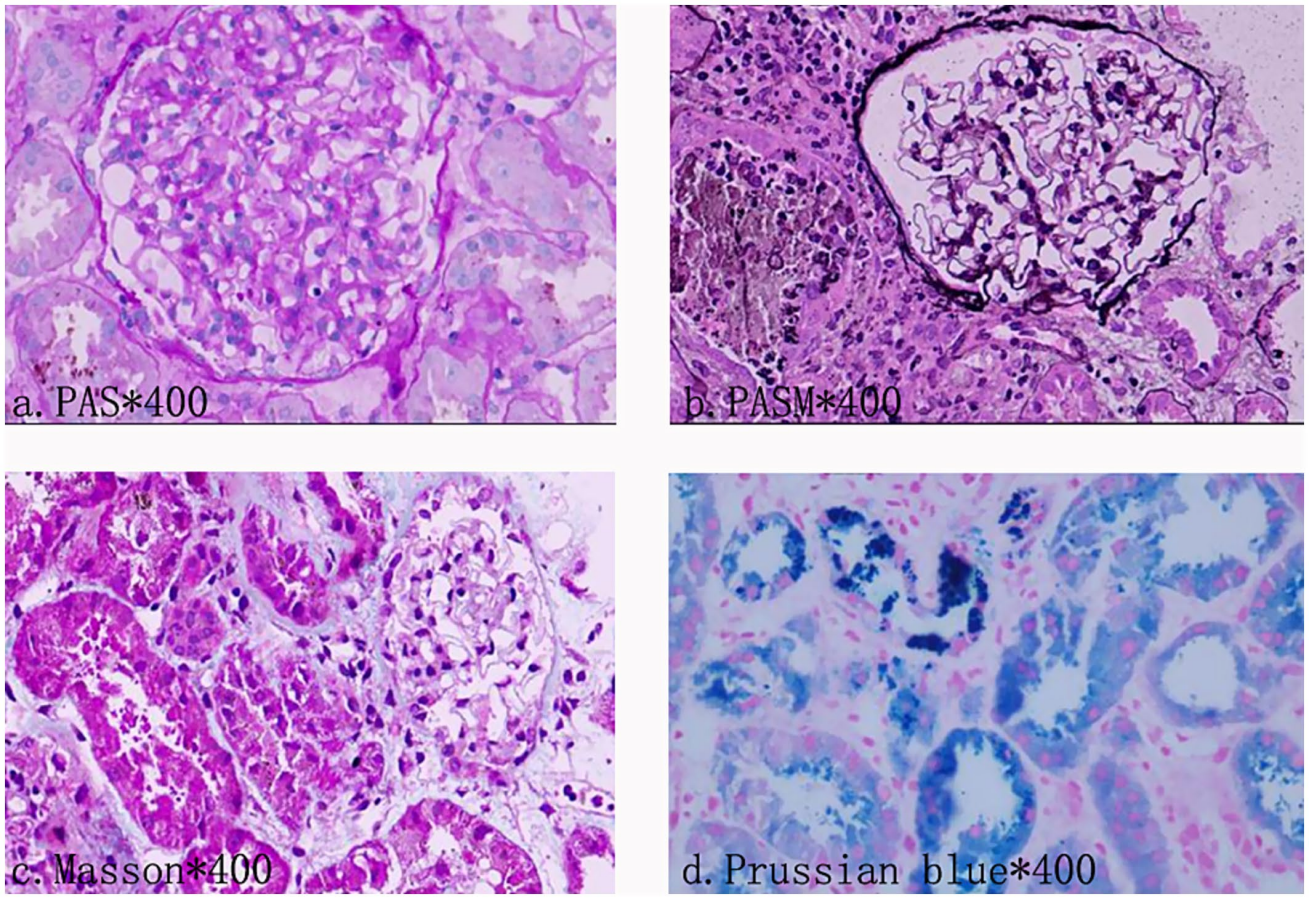
Photomicrograph of kidney biopsy. Light microscopy observations showed no glomerular sclerosis. Vacuoles and granular degeneration of renal tubular epithelial cells were observed. In the cytoplasm of the epithelial cells of the focal proximal renal tubules, hemosiderin, and a small amount of protein tube type can be seen. There are cellular components mixed in the tube type. There were some renal tubules expanded, and focal atrophy (about 5–10% of the atrophic area). Immunofluorescence examination documented no deposition of immune complexes in the glomerulus.

**Figure 2:**
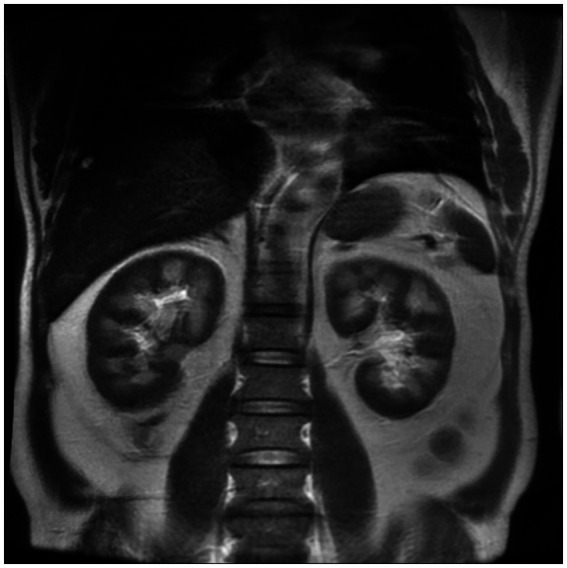
Renal magnetic resonance imaging. The renal MRI showed that the signal of the cortex on T1WI was lower than that of the medulla, showing a typical reversal of the renal cortex and medulla, suggesting that the cortex had manifestations of hemosiderin deposition.

**Figure 3:**
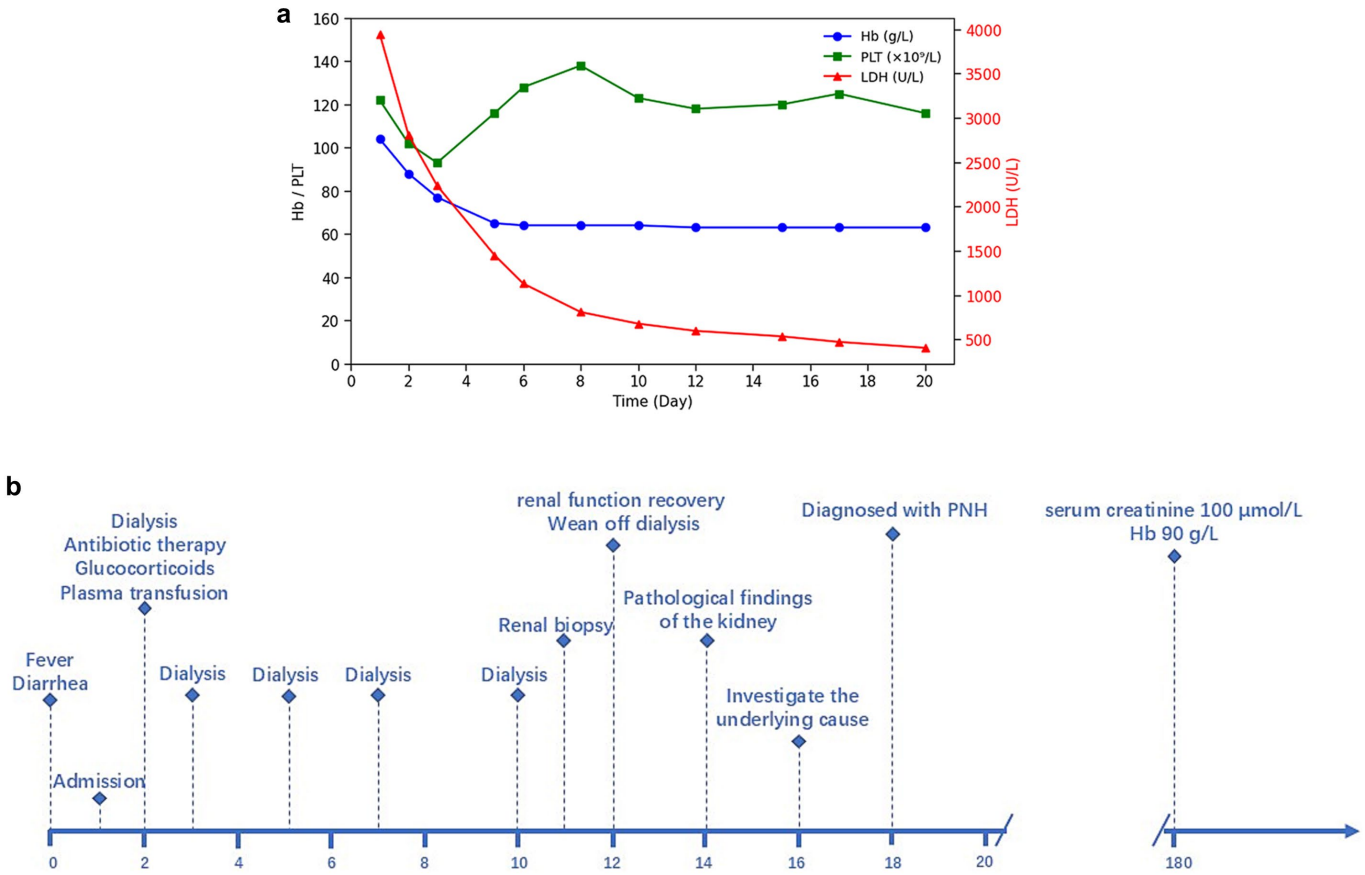
**(a)** Longitudinal changes of LDH, hemoglobin, and platelet counts over time. **(b)** Timeline of clinical events.

However, in the early stage of hemolysis and continuous deterioration of renal function, the platelet count remained stable after a slight decrease, which is not consistent with the typical clinical manifestations of aHUS. Therefore, to further elucidate the cause of AKI, a renal biopsy was performed. The pathological results (Figure 1) revealed the presence of hemosiderin in the cytoplasm of some proximal tubular epithelial cells, with no deposition of immune complexes in the glomeruli and nearly negative immunofluorescence. These findings suggest acute tubulointerstitial kidney injury associated with hemolytic anemia. To clarify the underlying disease, an extensive investigation into common causes of intravascular hemolysis was conducted. No abnormal cells were found in the bone marrow biopsy, which did not support the diagnosis of myelodysplastic syndrome or aplastic anemia. Tests for erythrocyte fragility, glucose-6-phosphate dehydrogenase, Ham test and urine hemosiderin were all negative. FLAER testing revealed a low level of PNH clones (7.8%) in red blood cells, including 0.5% of type II cells and 7.3% of type III cells, as well as 66.9% of granulocytes and 14.1% of monocytes. MRI showed enlargement of both kidneys, with higher T2-weighted imaging (T2WI) signals in the medulla compared to the cortex (Figure 2). The final diagnosis was PNH, and eculizumab treatment was recommended. However, the patient declined this option due to financial constraints and opted to continue with corticosteroid therapy, which was gradually tapered. The patient’s renal function improved, leading to the discontinuation of dialysis, and hemoglobin levels stabilized without transfusion dependence (Figure 3). During a 6-month follow-up, there was no recurrence of dark-colored urine, and renal function remained stable (serum creatinine 100 μmol/L), with hemoglobin levels around 90 g/L.

## Discussion

Intravascular hemolysis is a prominent feature common to both PNH and HUS. Although they exhibit similar clinical manifestations, their underlying pathogenesis differs significantly. PNH is a clonal hematopoietic stem cell (HSC) disorder characterized by hemolytic anemia, thrombosis, smooth muscle dystonias, and, in some cases, bone marrow failure. It results from a mutation in the PIGA gene, leading to the production of abnormal red blood cells deficient in CD55 and CD59 (1, 3). These cells become susceptible to destruction by the complement system, particularly during infections and other events that trigger complement activation, thus exacerbating paroxysmal hemolysis (4). Histological examination often reveals hemosiderin deposits in the proximal convoluted tubules as a common feature (5). According to previous literature, renal MRI can be used as a non-invasive method to visualize hemosiderosis in the renal cortex. Unlike other forms of hemolytic anemia, there is no iron deposition observed in other sites such as the liver or spleen (6). HUS is classified as a type of TMA and arises from abnormal activation of the complement alternative pathway, which leads to endothelial cell injury (2). The pathological manifestations include swelling and proliferation of endothelial cells, widening of the subendothelial loose layer, transparent tubular type and red blood cells in the renal tubules, thrombosis, and lumen stenosis in the arteriole (7).

The patient developed soy sauce-colored urine and acute renal failure following diarrhea, accompanied by anemia and a mild decrease in platelet count. The presence of erythroclasts suggests hemolytic anemia. Meanwhile, normal ADAMTS13 activity excludes TTP, thereby indicating a high likelihood of aHUS. Due to limitations in laboratory techniques, additional complement-related testing could not be performed to further substantiate the diagnosis. In light of the significant bilateral renal enlargement, which heightened the risk of bleeding during renal puncture, we initially initiated an empirical treatment regimen consisting of hemodialysis, corticosteroids, and antimicrobial therapy. Following this intervention, renal function was restored, and hemoglobin levels stabilized. However, certain clinical manifestations did not fully correspond with the typical presentation of aHUS. Consequently, we reconsidered the initial diagnosis and performed the renal biopsy, which failed to reveal endothelial damage or other hallmark features of TMA. Renal pathology, however, demonstrated hemosiderin deposition within the proximal tubules. During the investigation of the etiology of hemolytic anemia, the presence of abnormal PNH clones was identified. The negative result in the Ham test and the discrepancy in the PNH clone levels between red blood cells and granulocytes may be attributed to the sensitivity of the detection method and the timing of detection. The infection that occurred prior to testing led to activation of the complement system, resulting in exacerbated hemolysis. Recent hemolysis could reduce the proportion of GPI-deficient red blood cells, potentially obscuring the diagnosis of PNH (8, 9). The patient was diagnosed with acute tubular necrosis secondary to PNH induced by infectious factors. Additionally, the patient experienced a gradual decrease in hemoglobin levels following admission, which may be attributed to the infusion of fresh plasma. Prior studies indicate that activated complement in plasma, or complement activation through immune responses targeting platelet or leukocyte antigens, can enhance the lysis of PNH-cloned red blood cells (10).

Previous literature has documented that PNH can manifest with acute renal failure, particularly in patients with a history of hematological disorders (11). The patient in our case denies any history of hematologic disorders, which is relatively uncommon. Additionally, the lack of complement-related testing, combined with the risk of bleeding associated with renal biopsy, heightens the risk of misdiagnosis. The timing of the PNH-related examination, occurring post-acute hemolysis, led to atypical results in tests such as Ham test, urine ferritin, and other PNH-related assessments, further complicating the diagnostic process. When acute renal failure is accompanied by anemia, it is crucial to consider hematologic disorders as a potential underlying cause. Renal biopsy and additional diagnostic tests may be necessary to prevent misdiagnosis. Furthermore, infections can exacerbate hemolysis by activating the complement system, making the monitoring and control of infections crucial in the context of PNH.

## Learning points

- PNH is a rare cause of renal impairment, with atypical clinical symptoms that can lead to misdiagnosis as HUS or other hemolytic anemias. Enhancing understanding of PNH-related renal damage is essential.
- Clinicians should be vigilant for PNH in patients presenting with AKI accompanied by anemia and renal enlargement.
- Early differentiation can be achieved through flow cytometry, MRI, and kidney biopsy, thereby reducing the risk of misdiagnosis.
- In the absence of a clear distinction between PNH and TMA, plasma therapy should be used cautiously, as it may exacerbate hemolysis in PNH.

## Data Availability

The raw data supporting the conclusions of this article will be made available by the authors, without undue reservation.

## References

[1] Hill A DeZern AE Kinoshita T Brodsky RA. Paroxysmal nocturnal haemoglobinuria. Nat Rev Dis Primers. (2017) 3:17028. doi: 10.1038/nrdp.2017.28, PMID: 28516949 PMC7879566

[2] Michael M Bagga A Sartain SE Smith RJH. Haemolytic uraemic syndrome. Lancet (London, England). (2022) 400:1722–40. doi: 10.1016/S0140-6736(22)01202-8, PMID: 36272423

[3] Kokoris S Polyviou A Evangelidis P Grouzi E Valsami S Tragiannidis K. Thrombosis in paroxysmal nocturnal hemoglobinuria (PNH): from pathogenesis to treatment. Int J Mol Sci. (2024) 25:12104. doi: 10.3390/ijms252212104, PMID: 39596172 PMC11594924

[4] Macedo Ê S Parente Filho SLA Pro JDZ Rolim VM Primo GAS Brunetta DM. Renal involvement in paroxysmal nocturnal haemoglobinuria: a brief review of the literature. Rev Assoc Med Bras (1992). 2018;64:1139–1146. 10.1590/1806-9282.64.12.1139 30569992

[5] N MH Pinto CJ Poornima J Rajput AK Bagheri M Patil B. Classical paroxysmal nocturnal hemoglobinuria presenting with severe anemia and pigmented acute kidney injury. Cureus. (2022) 14:e28448. doi: 10.7759/cureus.28448, PMID: 36046061 PMC9417682

[6] Von Bodelschwingh B Ozkurt H. MRI as a Aiagnostic tool for paroxysmal nocturnal hemoglobinuria: a case report. Sisli Etfal Hastanesi tip bulteni. (2021) 55:565–8. doi: 10.14744/SEMB.2021.03443 35317371 PMC8907705

[7] Fakhouri F Zuber J Frémeaux-Bacchi V Loirat C. Haemolytic uraemic syndrome. Lancet (London, England). (2017) 390:681–96. doi: 10.1016/S0140-6736(17)30062-4 28242109

[8] Parker C Omine M Richards S Nishimura J Bessler M Ware R. Diagnosis and management of paroxysmal nocturnal hemoglobinuria. Blood. (2005) 106:3699–709. doi: 10.1182/blood-2005-04-1717, PMID: 16051736 PMC1895106

[9] Madkaikar M Gupta M Jijina F Ghosh K. Paroxysmal nocturnal haemoglobinuria: diagnostic tests, advantages, & limitations. Eur J Haematol. (2009) 83:503–11. doi: 10.1111/j.1600-0609.2009.01338.x, PMID: 19686268

[10] Laegreid IJ Wilson T Naess KH Ernstsen SL Schou V Arsenovic MG. Whole blood transfusion and paroxysmal nocturnal haemoglobinuria meet again: minor incompatibility, major trouble. Vox Sang. (2022) 117:1323–6. doi: 10.1111/vox.13354, PMID: 36102159 PMC9826352

[11] Nishimoto M Matsui M Tsushima H Tanabe K Tagawa M Samejima KI. Acute kidney injury in a postpartum woman with paroxysmal nocturnal hemoglobinuria: a case report and literature review. Hemodial Int. (2018) 22:E6–e10. doi: 10.1111/hdi.12591, PMID: 28796431

